# The Frailty Puzzle: Searching for Immortality or for Knowledge Survival?

**DOI:** 10.3389/fncel.2022.838447

**Published:** 2022-02-17

**Authors:** Stefano Govoni, Francesca Fagiani, Cristina Lanni, Nicola Allegri

**Affiliations:** ^1^Department of Drug Sciences (Pharmacology Section), University of Pavia, Pavia, Italy; ^2^CEFAT (Center of Pharmaceuticals Economics and Medical Technologies Evaluation), University of Pavia, Pavia, Italy

**Keywords:** aging, frailty, biological clocks, epigenetic clock, circadian clock, socio-economic factors, behavioral disturbances

## Abstract

What is the value of assessing the biological age and frailty and predicting residual lifespan and health status? The benefit is obvious if we have means to alter the pace of aging and the development of frailty. So far, limited but increasing examples of interventions altering the predicted status indicate that, at least in some cases, this is possible through interventions spanning from the economic-social through drug treatments. Thus, why searching for biological markers, when some clinical and socio-economic indicators do already provide sufficiently accurate predictions? Indeed, the search of frailty biomarkers and of their biological clocks helps to build up a mechanistic frame that may orientate the design of interventions and the time window of their efficacy. Among the candidate biomarkers identified, several studies converge to indicate epigenetic clocks as a promising sensitive biomarker of the aging process. Moreover, it will help to establish the relationship between personal aging and health trajectories and to individuate the check points beyond which biological changes are irreversible.

## Introduction

Undoubtfully, at planet level, humans are steadily increasing life expectancy and aging (United Nations [UN], 2020). Why is this happening and why are medical sciences so actively interested in counteracting frailty and fostering resilience? According to some historical, social and philosophical thinkers, we are in the process of designing a future in which humans will be immortal (Harari, 2016). On the other hand, the concept of preserving late life beyond active reproductive life and parental care period is counterintuitive for an animal species that, instead, should favor population growth, health and active reproduction. But changes took place since *Homo Sapiens* prevailed on other human species and evolved (Finlayson, 2010). In the blink of an eye, mankind have learned to deal with and control several events (*i.e.*, famine, war, plagues, also including Coronavirus Disease 19 (Covid-19) worldwide pandemics) that, in the past, could have led to human extinction (Harari, 2016), transitioning toward a knowledge-based society. No matter the existence of local cultural or political bursts of racism, we all share common ancestors and genome and an increasingly body of shared knowledge. A knowledge society (Stehr, 1994, 2018) has all the reasons to prolong life and intellectual life far beyond reproductive function, because even if cerebral competency, in particular in certain disciplines, peaks at relatively young ages, several other abilities develop throughout the entire lifespan and do not decline (Goldberg, 2006; Thomas et al., 2016). Richerson and Boyd (2020) underscore the role of adaptative cultural tradition processes that transformed mankind leading to an increasingly extended juvenile period spent to acquire social skills and to a post-reproductive survival prolonged period to transmit them. Indeed, a knowledge society cannot afford to lose accumulated experience. Consider, in a contemporary and different domain, the example of the importance of the knowledge generated by scientists, such as Steve Hawking, in spite of a devastating illness.

It might be that the present timeframe, during which aging-based life extension is considered important (beyond ethical, philosophical or religious considerations), may not last for long times overcome by other means of knowledge collection and evolution, as through AI or by programs of suspended life in the youth, while in the reproductive age necessary for interstellar exploration and traveling (Nordeen and Martin, 2019; Shi et al., 2021). But, presently the preservation of an active and, in particular, an intellectual active life is the reachable and most efficient way to outperform and attain our goal as species. Accordingly, the contrast of the age and illness associated frailty is a hot topic in biomedical research. It is matter of debate whether interventions at epigenetic, environmental or socio-economical level will be the most apt to contrast age-associated frailties and to promote resilience. It is an astonishingly complex biological puzzle. The following paragraphs will deal only with few nodal points, not pretending to be exhaustive, but just indicating some points of interest we came across while studying frailty associated with aging to further deepen and discuss in the scientific community.

## General Definition of Frailty

Frailty is a condition of increased vulnerability to interacting stressors, both endogenous and exogenous, resulting from the interaction of a progressive age-related decline in physiologic systems and disease-associated loss of functions, thereby leading to decreased functional reserve capacities (Clegg et al., 2013). The effect of frailty on patient’s clinical outcomes has been examined in several settings of care, including the recent Covid pandemics (Bellelli et al., 2020). In general, an increase in frailty predicts a worst outcome of the patients.

The Frailty Index (FI), or better Frailty Indices (FIs), since several of them have been proposed, are commonly used tools based on the concept that frailty is the result of an accumulation of deficits during the lifetime (Searle et al., 2008; Rockwood et al., 2015). The FIs evaluate tens of variables, including coexisting diseases, cognitive and physical impairments and laboratory abnormalities, suggesting that frailty results from failure of various systems. It is worth to underscore the importance that, in all frailty measures, is specifically given to motor control and muscle integrity. Indeed, motor activity and abilities have been studied among the first indices of frailty and biomarkers of sarcopenia have been considered linked to frailty and disability, taking into consideration the multifactorial nature of such phenomenon (Curcio et al., 2016).

Notably, sarcopenia which is present in average in half of the people over 80 years old, is associated in a decline in mitochondria quality control pathways resulting in mitochondria dysfunction of the sarcopenic muscle in turn releasing systemically signals (myomitokines, fibroblast growth factor 21 and growth an differentiation factor 15 influencing the whole body and the balance healthy/unhealthy aging (Romanello, 2020). Moreover, Picca et al. (2020) and Marzetti et al. (2020) showed that older adults with physical frailty and sarcopenia show increased levels of small extracellular vesicles and altered amounts of some mitochondrial components. These authors performed a multi-marker study exploring the relationship among systemic inflammation, metabolic derangements, and circulating mitochondrial derived vesicle in physical frailty and sarcopenia, and through a complex modeling defined five biomarkers [two amino acids (phosphoethanolamine and tryptophan), two cytokines (IL1-ra and MIP-1b), and a subunit of complex I of the mitochondrial electron transport chain (NADH:ubiquinone oxidoreductase subunit S3, NDUFS3)] able to discriminate older adults with and without physical frailty and sarcopenia. The authors further underscore the role sensed/played by the mitochondrial derived vesicle cargo in starting a chain of events bringing about systemic inflammation and further mitochondrial damage.

These aspects need more in depth studies also on the temporal relationship with the clinical signs, but are indeed indicating the possibility to detect new biomarkers as well as new targets for intervention. The consequences and the role of mitochondrial dysfunction may not be limited to sarcopenic muscle, but be involved also in other tissues including the brain and affecting neuronal function and regenerative abilities within a relationship between physical frailty and cognitive decline (Ma and Chan, 2020).

**FIGURE 1:**
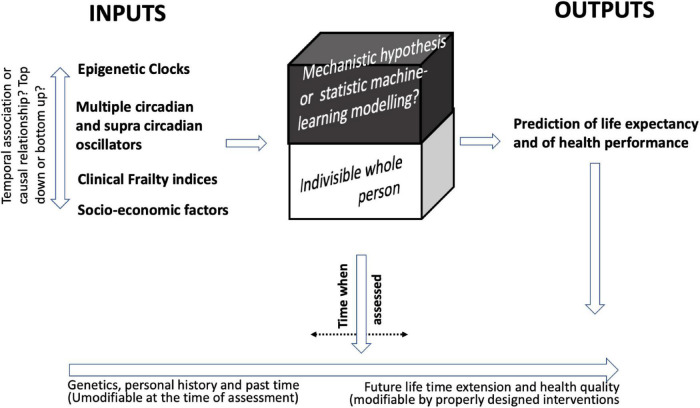
In search for frailty markers. The figure depicts the relationships between the multiple determinants of frailty and lists categories of indicators that have been studied as predictors of age and health in aged people. From a biological point of view, an important role is played by epigenetic clocks and circadian oscillators even if a hierarchical relationship between the two and among them and the clinical frailty indices and socio-economic factors cannot be established. On the other hand, the framework and the data collection generated by these studies let us predict the birth of increasing reliable predictor indices of residual life expectancy and health which may be assessed at various times of life trajectory. The so far available data also allow to predict that interventions able to change the pacing of these events may be developed. The figure highlights some aspects, such as: the dynamic nature of aging and frailty; the problem of identifying the relationship between biomarkers of aging and frailty in a complex system and the importance of the time-frame of the observation; the attempt to extend the biological frailty to other aspects of the life of older subjects including socio-economic aspects; the need of a multilevel approach which may rely also on the use of AI and machine learning to adequately select the relevant data and integrate them modeling an appropriate algorithm.

Biomarkers of sarcopenia and functional measures of muscle strength or velocity are an example of how an association between some individual markers and a functional measure is relatively easy to demonstrate. On the other hand, the important point is to establish the contribution of each measured activity/marker to the general picture (see also Figure 1 for a recapitulation).

In several cases both biological markers and clinical indicators are assembled in complex machine learning computations thanks to the widespread digital data, growing computing power, and advanced algorithms. Likely, in the next future, biological markers on accessible peripheral body fluids and frailty indices, based on easily performed clinical test, will be combined by using automated machine learning techniques to predict general, as well specific performances, in various domains at various time points, while aging, indicating tailored and time-framed specific interventions to sustain resilience.

Expanding the considerations on the concept of frailty it is worth to quote the work of Junius-Walker et al. (2018) who wrote a review dealing with the “essence of frailty” based on a systematic analysis of published frailty concepts and definitions. In particular the authors underscore that frailty does not fit in the traditional boundaries of “health and disease” and requires an approach based on the acceptance of the existence of complex systems based and integrating the interplay of biological and non-biological factors. The authors define 5 core components of frailty (Vulnerability, Genesis, Characteristics, Phenotype, Adverse health-related outcomes) and for each of them several defining and descriptive criteria. Within this context preventive and correcting interventions, including the evidence-based treatment of single conditions, have to be integrated in a health and care approach frame of reference. Such definitions and approaches increase the difficulty in defining the boundaries of normal aging and frailty and between the latter and the adverse outcomes as well as the role of comorbidities and disabilities. Also, within this context it will be important to recognize whether there are some biological events presenting pure aging-associated changes, independent from other life-events, which increase propensity to develop frailty in response to stressors and whether these changes can be prevented and counteracted. At the same time, it will be important to recognize whether the biological events underlying the various domains of frailty have common or specific “dedicated” determinants or a mix of the two. Moreover, also the biological correlates of the interaction between frailty and life events causing disability and death have to be explored in order to establish whether also these events can be prevented/reversed, allowing a healthier aging and postponing death. In other words where do the boundaries between reversible/irreversible changes lie in determining frailty and in transducing the interaction between frailty and life events (including but not limited to disease) into disability and death? So far, the research is still in the descriptive and speculative phase, many relationships have been delineated but the cause-effect and the sensitive time windows have not been conclusively defined yet.

## Epigenetic and Circadian Clocks

The theme of the biological epigenetic clocks within the context of aging and frailty indices and their interaction with circadian oscillators is intriguing. Taking into account that the circadian and supra circadian clocks/oscillators are not only merely instrumental to the daily living to synchronize physiological functions (sleep/wake-food intake-hormonal secretion-fertility cycles), can we consider their role in the organism aging process and its pacing? Samoilova et al. (2021) studied the interaction between circadian rhythms of gene expression and epigenetic clocks characterized by the specific profile of DNAm in CpG-islands, a parameter modified by aging. Azzi et al. (2014) first reported a direct role for DNA methylation (DNAm) in the circadian clock by exploring the effects of altered day lengths on clock plasticity. In particular, they found that, in mice, transient exposure to an altered lighting environment induced changes in global transcription as well as, in parallel, in promoter DNAm in the suprachiasmatic nucleus (SCN). Such results indicate DNAm as a potential mechanism regulating, at least in part, circadian clock plasticity and encoding a long-term signature of daylight on circadian function, thereby impinging upon several aspects of SCN neurophysiology. Consistently, in mice, stable methylation changes have been correlated with age-related alterations in circadian rhythms in certain peripheral oscillators (Zhang et al., 2013). However, the direct relationship with aging has to be fully understood yet.

Indeed, aging leads to a functional deterioration of many body and brain systems, including circadian clocks (Kondratova and Kondratov, 2012; Welz and Benitah, 2020; Acosta-Rodríguez et al., 2021). Their derangement seems to contribute to aging and age-associated impairment of adaptive functions (for example difficulties when changing time zone), and pathologies due to the altered circadian control of several processes including, for example, at cellular level: neuronal activity, immune responses, cell cycle, metabolism, redox homeostasis, hormonal secretion, autophagy, and stem cell proliferation. In some cases it has also been shown that interventions affecting health and life span, such as dietary restriction, may act through an action on energy and also the circadian system (as reviewed by Acosta-Rodríguez et al., 2021). Notably, epigenetic mechanisms may regulate circadian activity as shown for example in the case of SIRT1 which modulates the activity of several core clock genes (Asher et al., 2008; Nakahata et al., 2008).

However, it is not clear whether these changes occur due to the derangement of a central control or to an impaired brain-periphery communication and whether they consist in parallel changes or are hierarchically organized in a cascade; a combination of the two is likely. It is also unknown whether these clocks, based on the organized mutual control of brain and tissue-specific genes (Koronowski and Sassone-Corsi, 2021), will be reset by acting through external synchronizing interventions (for example food and bright light timing (Cuesta et al., 2017) and whether changes associated with a pathology will be differently responding compared to age-associated changes in absence of pathology.

In rodent models, as reviewed by Panagiotou et al. (2021), it has been demonstrated that even moderate age-matched exercise is able to ameliorate several aging features, as far as sleep and circadian rhythms are concerned, independently from the species under investigation. It should be underscored that the technological nature of modern society, with round-the-clock work schedules has had a negative impact, leading to an increase in the incidence of circadian and sleep disorders. Interestingly, De Nobrega and Lyons (2020) working on a simple organism, the *Drosophila melanogaster*, on the basis that aging and circadian interactions are conserved evolutionary aspects, were able to show the complex activity of a central master circadian clock responsible for synchronizing many tissue specific circadian oscillators in a hierarchical manner.

Although differences in individual genes exist between the core oscillators of *Drosophila* and mammals, the underlying mechanisms of clock function are similar. Transcription factors in the positive limb activate transcription of genes in the negative limb that then repress the transcriptional activators originating the period. Beyond the apparently easy mechanism it should be recalled that in a study of the effect of aging on circadian gene expression in human brain tissue, circadian rhythmicity has been found altered in an age-dependent manner for approximately 1,200 genes with changes in rhythmicity found for genes involved in cognition, sleep and mood regulation (Chen et al., 2016).

## Time and Biological Variables: What Is the Real Predictive Value of Biological Markers?

The search for biomarkers of aging and frailty is sustained by the observation of the highly heterogenous inter-individual variations between subjects of the same chronological age. The identification of biomarkers with a predictive value of aging process and frailty and accounting for the individual differences is critical to establish therapeutic interventions aimed to slow down the aging clock and to contrast frailty, before the onset of irreversible biological changes. Among the candidate biomarkers identified, several studies converge to indicate the epigenetic clocks as a promising sensitive biomarker of aging process. Accordingly, research on the epigenetic clocks measuring changes in DNAm at specific Cytosine-phospho-Guanine (CpG) sites showed a differential methylation of specific genomic locations of CpG according to age, accounting for biological age acceleration (AA)/deceleration. However, the first generation epigenetic clocks simply based on calendar age were weakly correlated with functional health and/or cognitive/neuropsychological performance. Successive studies (McCrory et al., 2021) working on second generation epigenetic clocks (e.g., PhenoAge and GrimAge) examined the association of epigenetic clocks with clinical phenotypes by including DNAm correlates of morbidity and mortality (Levine et al., 2018; Lu et al., 2019). In particular, McCrory et al. (2021) found that, in a sample of 490 participants belonging to the Irish Longitudinal Study on Ageing (TILDA), one of them (i.e., GrimAgeAA) was associated with several clinical relevant parameters, including walking speed, Fried frailty, polypharmacy, Mini-Mental State Examination (MMSE), Montreal Cognitive Assessment (MOCA), Sustained Attention Reaction Time, 2-choice reaction time, and with all-cause mortality at up to 10-year follow-up. While still in its infancy, the validation of the second-generation clocks suggest that they may be able to predict a range of important age-related outcomes and life span resulting in better marker of premature aging and predictors of age-related decline in clinical health measures. However, whether DNAm is a cause or a consequence of aging is still matter of scientific debate and needs further investigations to measure changes in both health and patterns of DNAm over the time in order to establish the temporal sequence of the observed relationships. Moreover, the observation that epigenetic signatures during aging may be modified by treatments (see the data on mice, as well as in humans (Fahy et al., 2019; Schultz et al., 2020) makes even more interesting the search of the causal relationships between the marker and the clinical phenotype.

On the other hand, the link between biological markers and indices of frailty is still matter of an open debate. For example, Bacalini et al. (2021) demonstrated that, in a group of Italian semi super centenarians (over 105 years old), the epigenetic clock represented by DNAm, considered as representative of the biological age of the individual, did not correlate with the frailty index (consisting of a series of quantitative evaluation of the age-related accumulation of health deficits). The latter, on the other hand, is able to predict mortality and health conditions, and discriminates different degrees of frailty, even at very advanced age (see also Gu and Feng, 2015; Arosio et al., 2019). Overall, these data indicate that it is possible that clinically based frailty indices and epigenetic clocks may be associated with different aspects of the aging process within the same individual.

Obviously, these scientific considerations have also important ethical and regulatory implications. As an example, a life insurance company may start predicting the cost of a patients as a function of evidence-based frailty indices. Indeed, it has already been shown (Johnston et al., 2020) that a model including the frailty index resulted in more accurate predictions of costs for patients at various levels of frailty. Within this context, Diebel and Rockwood (2021) have proposed that grading of frailty is a clinically and research-relevant estimate of biological age and that emerging biomarkers can supplement this approach by identifying accelerated aging before it is clinically apparent.

## The Contribution of Preclinical Data in Animal Models

An extraordinary experiment on the frailty index value is that performed by Schultz et al. (2020) built by measuring in mice from 21 months of age till death a series of parameters and generating by machine learning two predictive clocks FRIGHT (Frailty Inferred Geriatric Health Timeline) clock, a strong predictor of chronological age, and AFRAID (Analysis of Frailty and Death) clock, which accurately predicts life expectancy and the efficacy of lifespan-extending interventions.

In their work the authors argue that the DNAm clocks are correlated with the chronological age, but they require repeated invasive blood collection and more complex procedures compared to the other functional markers they adopted (for the complete list of the 32 considered parameters -from tail stiffening to nasal discharge - see Table 2 in the cited paper). Moreover, they predicted individual life expectancies rather than increased risk of mortality at population level, suggesting their AFRAID clock could be adapted also to human studies, thereby providing the collection of large datasets of longitudinal assessments.

Once again, the debate between biomarkers as measurable biochemical parameters in accessible body fluids and biological markers as extensive analysis of both biochemical and/or functional activities has not been solved yet. The impression is that biochemistry-based parameters will predict trajectories within more specific domains, whereas more integrated parameters and behaviors will gain a greater systemic predictive value, but this hypothesis has to be still tested. Indeed, clinical related parameters may recapitulate the integrated results of several interacting processes acting in parallel rather than in an ordered serial sequence.

The animal models do mainly contribute allowing, in a convenient time frame and in controlled situations, to evaluate definite aspects of the phenomenon as well as of substances negatively or positively affecting it. However, it is clear that the information stemming out from animal models may only provide valuable suggestions that have to be then verified in the clinical setting in humans.

## Are Socio-Economical Aspects Impinging Upon Biology of Frailty?

Let us ask a direct question: how much does our personal social life trajectory influence our biology? It sounds obvious that poverty and poor social life are associated with loss of abilities to cope with stressors and be resilient. However, the link appears even more strict than originally proposed. Indeed, Simons et al. (2016) were able to show using structural equation models and longitudinal data from a sample of 100 Black, middle-aged women residing in the United States, the effect of income on a developed epigenetic measure of biological aging that is DNA methylation. Most importantly the authors showed that low income displayed a robust association with accelerated aging unaffected by other commonly considered factors such as education, marital status, and childhood adversity. According to the authors the analyses indicated that the association between income and biological aging was not explained by unappropriate health-related behaviors (diet, exercise, smoking, alcohol consumption). Rather, in large measure, it was financial pressure that accounted for the association between low income and accelerated aging.

The effect of the socio-economic status (SES) and epigenetics has been further explored by Martin et al. (2021) exploring epigenetic clocks (Horvath, Hannum, and PhenoAge) and showing that living in adverse neighborhood environments is associated with accelerated DNAm aging. In particular DNAm aging was elevated in neighborhoods with lower social cohesion, whereas positive neighborhood attributes were buffering the effects.

Interestingly enough, Cui and Chang (2021), using the panel data from the 2011, 2014, and 2017 waves of the Chinese Longitudinal Healthy Longevity Survey (CLHLS) pointed the attention to the fact that relative income, rather than absolute income, has a significant negative impact on health performance, and that these associations may be causal in nature. The effect persists throughout the life cycle, which shows that, even in the old-age stage, the influence of external socioeconomic factors on health still transcends that of individual biological factors. In their research they used Self-rated health which is a somewhat subjective, but also an inclusive and accurate indicator.

The view that frailty is not only a physical problem, but also refers to emotional, social, and environmental hazards is underscored by the work of Dury et al. (2017) in Belgium evaluating sociodemographic and socioeconomic indicators as well as four dimensions of frailty (physical -for example, walking upstairs -, psychological - mood disorders and emotional loneliness -, social -presence of social support -, environmental – *e.g.*, suitability of the physical housing environment- domains). Their findings indicated that, for both men and women, increased age, having no partner, having moved to a new house in the previous 10 years, having a lower educational level and having a lower household income are risk characteristics for frailty.

The quoted papers, even if based on different methodologies and samples, suggest relevant considerations involving policy makers and administrators. Medical sciences may provide good points in terms of personal lifestyle (attention to a healthy diet, exercise avoid smoking, alcohol, abuses etc.). However, this may not be sufficient if not accompanied by proper social intervention and policies of equity in income distribution. The interventions are really complex when trying to put into the equation health. In this regard, the paper by Cui and Chang (2021), focused on the Chinese society, well underscores that pushing the country economy to elevate people’s income standards is one of the leverages, but at the same time increasing personal work load and stress, environmental effects and reduced time for leisure may have negative health effects, therefore interventions have to be very carefully balanced.

In any case the strength of the associated biological variables when measured is surprising and underscores the fact that what we generally consider independent life variables instead profoundly affect our biological setting beyond just having enough food, water and energy to sustain basic survival.

## Gratification and Frailty

The above depicted view is supported also by observations on the relationship between gratification and frailty. Thinking and feeling well following being gratified for having done a proper work or accomplished a task improves the health status. Jackowska et al. (2016) compared the effect of a gratitude intervention (highlighting the positive events of the day) to an active control (simple everyday events reporting) in 119 young women. The treatment elicited increases in hedonic well-being, optimism and sleep quality along with decreases in diastolic blood pressure. The intervention lasted for a brief two weeks period, suggesting that, in spite of the short period of observation, subjective well-being may significantly contribute toward healthier biological function and restorative health behaviors. No frailty measures were performed in the study and all the participants were young. However, it can be speculated that similar interventions may affect frailty parameters as suggested by Ahmed et al. (2021), discussing behavioral change techniques (see also the next paragraph). Also, by examining the association between dispositional optimism compared to dispositional pessimism and ideal cardiovascular health, Serlachius et al. (2015) found that low pessimism was associated with ideal cardiovascular health, especially with health behaviors. Moreover, Sardella et al. (2021) have shown that dispositional optimism (belief that the future generally holds positive, but not negative events) appears to confer widespread benefit in terms of psychosocial well-being. In particular, the subjects who exhibited a better health status also had higher levels of dispositional optimism and better global cognitive functioning.

These views are further supported by the work by Gale et al. (2014) investigating whether psychological well-being was associated with incidence of physical frailty. By studying a sample of over 2,500 men and women over 60 years old from the English Longitudinal Study of Aging, they found that higher levels of psychological well-being were associated with a lower probability to become frail over the 4-year follow-up period. Examination of scores for hedonic (pleasure) and eudemonic (control, autonomy and self-realization) well-being showed that higher scores on both were associated with decreased risk by mechanisms yet unidentified.

## Behavioral Disturbances and Frailty

Within the proposed general reference frame, all the aspects contributing to frailty are of obvious importance, but a priority is given to those affecting cognition. From a general point of view, Zijlmans et al. (2021) showed that, in a population-based sample of older adults, higher cognitive and brain reserves (not simply years of education) were associated with a lower risk of mortality. Changes in cognitive abilities in the aged population are popularly associated with memory complains and deficits that are not necessarily the first and more important changes. Rather behavioral disturbances (54) (Fan et al., 2020) or deficits of the executive function may have a greater importance and are frequent also in otherwise healthy aged people under stress conditions, such as hospitalization for an acute illness. Within this context Semprini et al. (2012) highlighted the importance of impaired cognition as factor predisposing to frailty suggesting that the presence of neurobehavioral disturbances, such as apathy, associated to impaired executive function, could have a major role in predisposing for frailty and unsuccessful aging. This is an aspect largely underestimated in the literature and without recognized clear-cut biological markers. Likely, considering *ex adiuvantibus* criteria, *i.e.*, responses to drug treatments, some of these behavioral disturbances may rely on primary or secondary classical neurotransmitter defects present in aging and also in the prodromic phases of neurodegenerative diseases contributing to early neurobehavioral alterations matching the clinical definition of mild behavioral impairment (Ismail et al., 2017). Interestingly, Lussier et al. (2020) demonstrated a link between Mild Behavioral Impairment (MBI) and early AD pathology, in particular beta-amyloid deposition in brain, in a cognitively intact elderly population. Also, tau pathology has been associated by others (Johansson et al., 2021) with MBI independently from memory deficits. The above considerations suggest that two of the proteins that have been considered as central players of AD pathology may be rather associated with behavioral and cognitive disturbances preceding the neurodegenerative stadium. Such evidence indicates the importance to investigate them, also in aging and other dementias, at least in the case of beta amyloid which, compared to tau, shows a more net age-associated pattern in non-AD subjects (Rodrigue et al., 2009).

Within this context, Fan et al. (2020) showed that both frailty and MBI status were associated with higher odds of cognitive impairment. Moreover, MBI was significantly associated with an increased risk of frailty in the absence of dementia. It is obvious the importance of an early detection of this condition also to plan prevention and intervention actions to reduce its impact on frailty.

These observations underscore the importance of a thorough neuropsychological assessment and evaluation of behavioral disturbances and of the search of appropriate biological markers to better identify risk markers of frailty.

On the other hand, general attention to the behavior and training on appropriate behaviors may have positive effects, as suggested by Ahmed et al. (2021), showing that behavior change techniques in personalized care planning for older people may improve the quality of life of old people. From the meta-analysis performed by the authors, six behavioral change techniques emerged including, ‘goal setting’, ‘action planning’, ‘problem solving’, ‘social support’, ‘instructions on how to perform a behavior’, and ‘information on health consequences’. Several of these are directly related to cognitive and executive abilities and others toward social support, once again supporting the idea that aiding cognitive functions may favorably impinge upon frailty. These concepts are also driving national initiatives such as Ageing Well National Science Challenge (2021) having strategic targets as reducing social isolation, promoting social connection and building connections.

## Conclusion

Some of the observations on the relationship between multiple determinants of frailty have been schematically represented in Figure 1. It is a partial view since the list of the age-associated clinical and biological parameters which have been used for frailty indices is, to date, almost endless (Rivero-Segura et al., 2020; for a categorization and an analysis see Lohman et al., 2021). Among those not here considered, just to mention some markers that have been thoroughly investigated, are the age-associated changes in the immunological (Fahy et al., 2019; Sayed et al., 2021) and in the inflammatory responses which are somewhat linked (Batista et al., 2020), in oxidative stress/antioxidant reserves (Kameda et al., 2020) or telomer length (Vaiserman and Krasnienkov, 2021). Likely the future development of frailty and longevity indices, able to predict residual life expectancy and health, will rely on the composite use of several clinical and biological markers optimized using machine learning programs. These tools will be also useful in the process of evaluating pharmacological and non-pharmacological interventions aimed at correcting accelerated pacing (Lohman et al., 2021). Studying time-related frailty biomarkers imposes another set of considerations. Maestrini et al. (2018) suggest that relativistic corrections of physical time similar to those used in the Einstein relativistic theory resulting from accelerated or decelerated biological dynamics may be used to model the pace of aging. Such theoretical framework places and describes the biological dynamics as trajectories in a biological space-time reference system. The work underscores the importance of a frequently neglected parameter in most of the biological experiments that is time and domains (space) of influence and the different pacing of various coexisting biological events and their conditioning of time and space events in related system, as well as the resultant effect at organism level. The proposed theoretical framework may help to better put in a hierarchical order the events, their respective space-time domains, improving the approach to the aging studies. In other words, a better understanding of the intrinsic space-time relationship in different specific events may help an external observer to describe the system pacing at organism level and to understand how external events may introduce synchronization/desynchronization.

An important question that needs to be answered is whether the biological changes anticipate the clinical ones or not. Wang et al. (2019) in their analysis of the studies examining selected biological processes and biomarkers related to frailty in older adults found bidirectional relationships between and within the various processes and a variable strength of the association of the biomarkers with an unclear causality. The multifactorial nature of frailty, its sensitivity to socio-economic and disease variables, and the reversibility of the process in certain conditions do suggest that it will not be easy to distinguish those processes that are preceded by appreciable changes of the biomarkers and those that are accompanied and just associated with an index biomarker change useful to size the effect but not to predict it. Moreover, Verghese et al. (2021) in a prospective cohort study on 681 older adults showed that different frailty trajectories exist (relatively stable, mild, moderate and severely frail) with a different pacing in accumulating additional deficits. The frailty trajectories were distinguished by clinical and proteomic measures at baselines (11 proteins of the 4,265 measured). Finally, it should be stressed that some studies on centenarians describe a dissociation between the biological frailty indexes and the clinical ones (Arosio et al., 2019, 2021; Bacalini et al., 2021) suggesting that in very old people the relationship between chronological age, biological age and clinical status needs to be further studied. Accordingly, frailty biological indices need to be validated by multiple measurements during the aging process as well as the strength of their association/predictive value with the clinical status.

Within this reference frame, both at conceptual and experimental level, it will be important to explore which processes are resultant from a hierarchical organization amenable to a triggering event and which ones are the consequence of parallel events and therefore need multitargeting of the designed intervention. Another important aspect will be to examine the importance of the time sampling of the adopted parameters. As shown in the figure, the time of assessment can be a mobile window. Reassessment will be an important tool to evaluate the effect of the adopted interventions and also to establish the time-window of the effectiveness of an intervention beyond which changes are not any more reversible.

Finally, it will be also relevant to understand how much of the daily life data communicated through the social media, beyond clinical data collection, will be useful if included in the analysis. Notably, a possible evolution of neuropsychological testing and helping is consisting of gamification of the tests using smartphone Apps (for a critical analysis of this field see Edwards et al. (2016), it is even possible that future AI based analysis of the social web pages of people will be able from their stories, language and networking level to extract FI and cognitive profiles even from unstructured information, see for example the EU project FrailSafe (2021). Accordingly, attention should be paid to the ethical, legal and privacy limits that as a society we are willing to establish to the free circulation of what may become a very sensible information from an apparently trivial string of text.

## Author Contributions

SG and NA conceived the presented idea. CL and FF investigated specific aspects related to circadian oscillators and DNA methylation. All the authors then discussed the integration of the various sections of the manuscript and their final sequence.

## Conflict of Interest

The authors declare that the research was conducted in the absence of any commercial or financial relationships that could be construed as a potential conflict of interest.

## Publisher’s Note

All claims expressed in this article are solely those of the authors and do not necessarily represent those of their affiliated organizations, or those of the publisher, the editors and the reviewers. Any product that may be evaluated in this article, or claim that may be made by its manufacturer, is not guaranteed or endorsed by the publisher.
